# Primary bone lymphoma: A case report and review of the literature

**DOI:** 10.3892/ol.2014.2327

**Published:** 2014-07-09

**Authors:** HAI-YAN ZHOU, FANG GAO, BING BU, ZHENG FU, XU-JIE SUN, CHENG-SUO HUANG, DENG-GUANG ZHOU, SHU ZHANG, JUN XIAO

**Affiliations:** 1 Internal Depatment of Oncology, Shandong Cancer Hospital and Institute, Jinan, Shandong 250117, P.R. China; 2 Positron Emission Tomography-Computed Tomography Center, Shandong Cancer Hospital and Institute, Jinan, Shandong 250117, P.R. China; 3 Department of Pathology, Shandong Cancer Hospital and Institute, Jinan, Shandong 250117, P.R. China

**Keywords:** primary lymphoma of bone, diagnosis, therapy, prognosis

## Abstract

Primary lymphoma of the bone (PLB) primarily arising from the medullary cavity is an extremely rare entity, with only retrospective studies and sporadic cases reported in the literature. The current study presents one case of PLB treated with chemotherapy and radiotherapy, and a review of the literature to elucidate the optimal treatment of PLB. A 73-year-old female presented with pain in the left hip that had persisted for two months. Plain X-ray and magnetic resonance imaging of the left hip showed lytic areas involving the left innominatum. Technetium-99m radionuclide imaging showed increased tracer uptake in the ilium, acetabulum and ischium. An ^18^F-fluorodeoxyglucose-positron emission tomography-computed tomography (FDG-PET-CT) scan showed high FDG uptake. A fine-needle aspiration biopsy of the lesion was performed, and histopathological and immunohistochemical examination confirmed a diagnosis of B-cell lymphoma. The patient received radiation therapy followed by six cycles of CHOP regimen (1,000 mg cyclophosphamide, 80 mg epirubicine and 2 mg vincristine on day one, and 100 mg prednisone on days one to five, every three weeks) and achieved a complete response, as confirmed by FDG-PET-CT. At present, the patient is in a good condition. This case is noteworthy, as it is a well-documented case in which the patient received successful treatment. This case demonstrates that PLB has an improved prognosis compared with primary lymphoma of other sites; however, combined therapy may further improve the patient outcome.

## Introduction

Primary lymphoma of the bone (PLB) is an extranodal lymphoma that arises from the medullary cavity and manifests as a localized, solitary lesion, which represents ~3% of all primary malignant bone tumors and 1% of all malignant lymphomas (1). PLB was first described by Oberling in 1928 (2) and is generally an extremely rare condition. The cause of PLB is not well-known and any part of the skeleton can be involved (3). The cell subtype of PLB varies and the molecular features have not been well studied (4). Staging varies with different diagnosing criteria at different times (5). Imaging features are usually non-specific (6). As PLB is a highly curable disease, it is important for it to be differentiated from other causes of lytic bone lesions, such as carcinomas and other primary bone tumors. The prognosis of PLB improves following chemotherapy and radiotherapy. The present study reports one case of PLB of the bone and a review of the literature with regard to PLB to elucidate the clinical manifestation, imaging features, staging, diagnosis and differential diagnosis, optimal treatment and prognosis of this unique disease. Patient provided written informed consent.

## Case report

**Figure 1:**
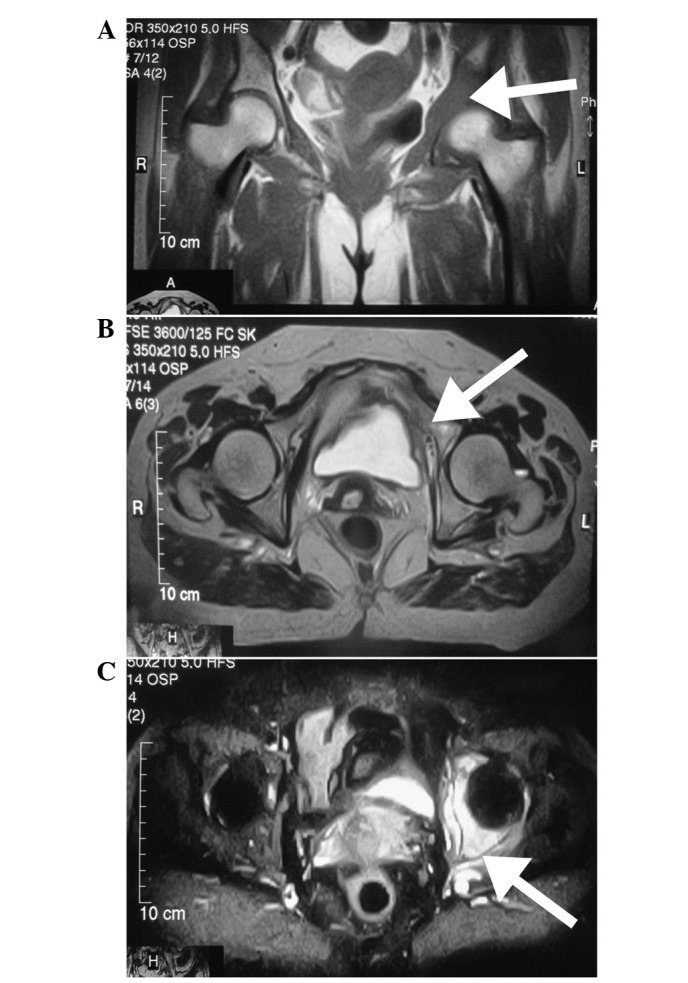
Magnetic resonance imaging showing cortical disruption of the acetabulum and a soft-tissue formation. The signal intensity of the soft-tissue section was (A) mostly homogeneously hypointense (arrow) compared with the bone on T1-weighted images, (B) isointense or slightly hyperintense (arrow) on T2-weighted images and (C) hyperintense (arrow) on short TI inversion recovery.

A 73-year-old female presented to the Internal Department of Oncology, Shandong Cancer Hospital and Institute (Jinan, China) with pain in the left hip that had persisted for two months. Plain X-rays showed no abnormalities of the pelvic bones, however, magnetic resonance imaging (MRI) of the left hip was performed and showed abnormal signals involving the left innominatum, with soft tissue formation. The signal intensity was low on T1-weighted images (Fig. 1A), high or isointense on T2-weighted images (Fig. 1B) and hyperintense on short TI inversion recovery (Fig. 1C).

**Figure 2:**
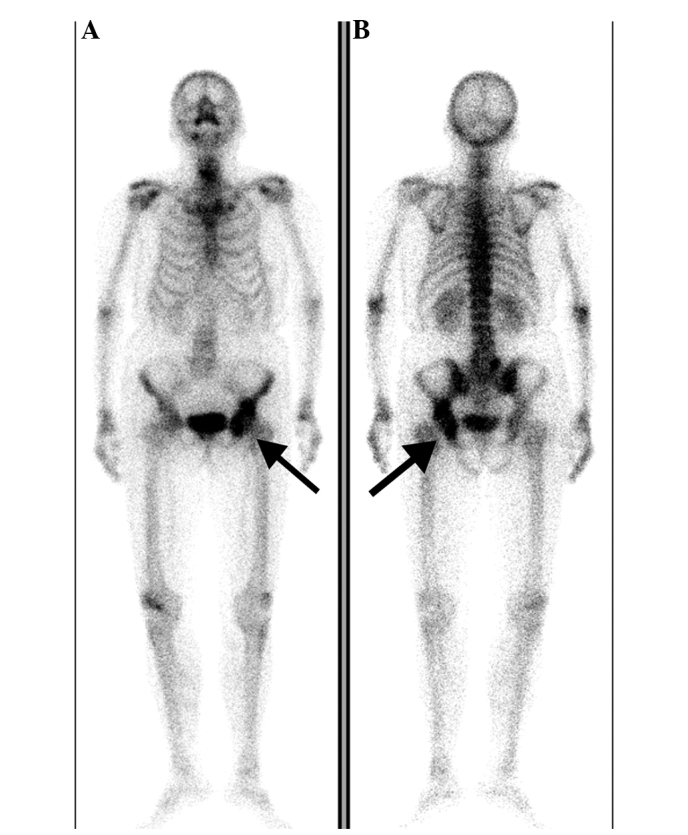
(A) Anterior amd (B) posterior radionuclide bone scans showing an abnormally high distribution of technetium-99m in the left innominatum (arrow).

Technetium-99m (^99m^Tc) radionuclide bone scans were performed to rule out multiple bone lesions, and increased tracer uptake was shown in the left innominatum, including the ilium, acetabulum and ischium (Fig. 2).

**Figure 3:**
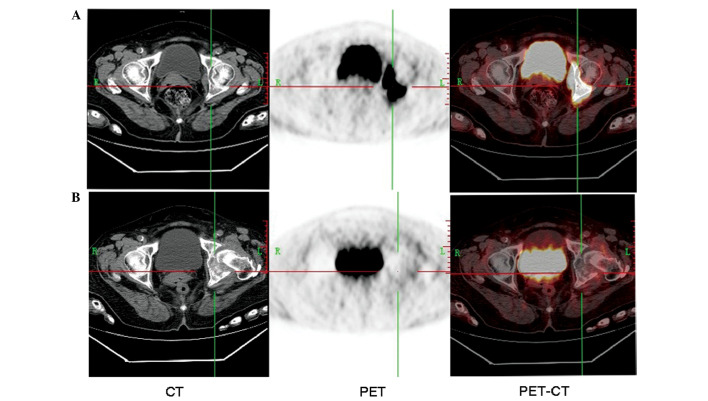
FDG-PET-CT scan of the whole body. (A) Prior to treatment, FDG-PET-CT scan showed an abnormally high uptake of ^18^F-FDG in the left acetabulum. (B) FDG-PET-CT scan following treatment showed no hypermetabolic lesions in the left acetabulum, however, the neck of the left femur was broken, with displacement of the distal section. FDG, fluorodeoxyglucose; PET, positron emission tomography; CT, computed tomography.

An ^18^F-fluorodeoxyglucose-positron emission tomography-computed tomography (FDG-PET-CT) scan was performed to identify the original site of the tumor. Abnormal ^18^F-FDG uptake was found in the left innominatum, with a peak standardized uptake value of 60.7, lytic lesions and soft-tissue lump formation ~7.8×4.5×8.8 cm in size (Fig. 3A).

**Figure 4:**
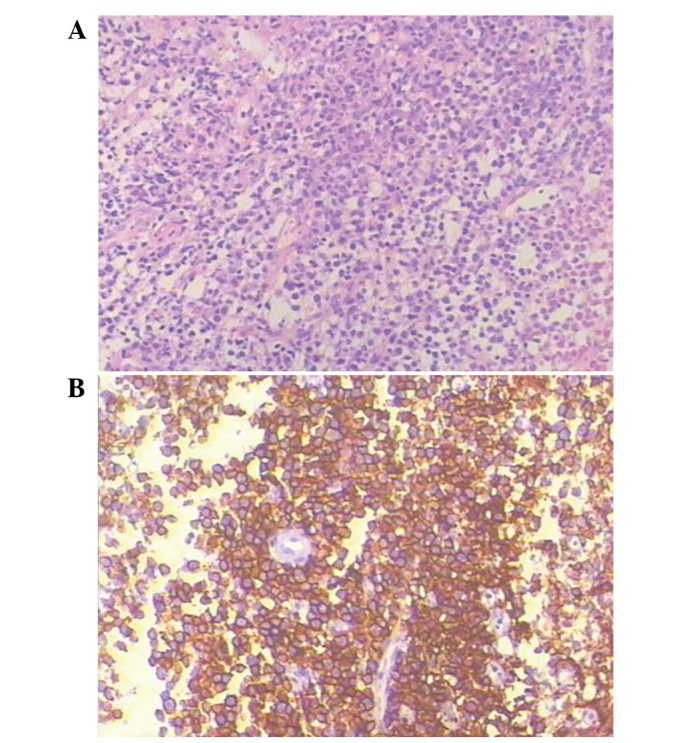
(A) Histopathological examination of a hematoxylin and eosin-stained section showed diffuse, round tumor cells of approximately the same size. (B) Immunohistochemistry showing strongly positive staining for cluster of differentiation 20 in the cell membrane. Original magnification, ×100.

Next, a fine-needle aspiration biopsy of the lesion was performed and a histopathological examination showed diffuse, round tumor cells of approximately the same size. Immunohistochemistry showed that the cell membrane was strongly positive for cluster of differentiation (CD)20 and negative for CD79α, CD3, CD78, CD138, cytokeratin (CK) and CK8/18, supporting a B-cell origin (Fig. 4). Further examinations, including bone marrow aspiration and biopsy, and CT scans of the neck, chest and abdomen, were normal.

The patient was diagnosed with PLB of the left innominatum, classified as stage I–E according to the Ann Arbor system (5). The patient was treated with 50 Gy radiation therapy in 25 fractions over five weeks, followed by six cycles of CHOP regimen (1,000 mg cyclophosphamide, 80 mg epirubicine and 2 mg vincristine on day one, and 100 mg prednisone on days one to five, every three weeks). Following the third cycle of CHOP, the patient experience abnormal pain in the left hip. The FDG-PET-CT examination was repeated, but no abnormal FDG uptake was observed (Fig. 3B). However, the neck of the left femur was broken, with displacement of the distal section. At present, the patient is being regularly followed up and has remained disease-free since the last treatment, with the exception of the broken femoral neck.

## Discussion

The cause of PLB is not well-known now, however, viral infection, immunodeficiency, organ transplantation, Paget’s disease of the bone and inherited factors have been identified as possible causes in the process; although this has only been found in retrospective studies (7). The majority of PLB patients are >45 years of age and there is a slight male preponderance, with a male to female ratio of 1.2:1.8 (5). Involvement of any region of the skeleton is possible, however, a trend exists in favor of the long bones with persistent bone marrow (3). The most commonly affected site is the femur, which accounts for ~50%, with tumor cell infiltration along the shaft of the bone longitudinally. The pelvis is the secdonary affected site with a proportion of ~20%, while other sites include the spine, ribs, mandible, scapula and proximal phalanx of the thumb (8). PLB differs from secondary lymphoma of the bone, where the axial bones are the most common sites of presentation. Furthermore, certain clinical characteristics of PLB in the Asian population differ from those in Western populations, with the pelvis being the most commonly involved site (52%) (9–11). Patients with PLB commonly present with local bone pain, soft-tissue swelling, a mass or pathological fracture, or hypercalcemic crisis (12). ‘B’ symptoms are rare and are only observed in stage IV patients (13).

The staging of PLB varies with the diagnostic criteria as this changes over time. According to the Ann Arbor system (3), currently the most widely accepted staging system, PLB is divided into the following four stages: i) Stage I, single lesion in the bone with or without soft-tissue infiltration; ii) stage II, more than two lesions beside one side of the diaphragm, or a single lesion in the bone with soft-tissue infiltration; iii) stage III, lesions beside two sides of the diaphragm; and iv) stage IV, infiltration of the central or peripheral nervous system, or bone marrow, as determined by staging biopsy at different times.

According to the Ann Arbor system, when a full staging evaluation is performed, the majority of patients exhibit stage IE or IIE disease (7,10). A study by Heyning *et al* (11) classified 46% stage I, 16% stage II and 16% stage IV PLB patients and 20% with an unknown stage. Stage IV disease was exclusively caused by the presence of multiple bone lesions (11). In a prospective study that included 28 PLB cases, the Ann Arbor stage distribution was 54% for stage I–II and 46% for stage III–IV (14). Other studies have also obtained similar results, with disease stages IE or IIE constituting the majority of PLB cases (13,15,16).

Non-Hodgkin’s lymphoma (NHL) forms the majority of PLB, with the most common subtype being of B-cell origin (4,17) whereas primary Hodgkin’s lymphoma of the bone is extremely rare (7). The B phenotype constitutes 78–100% of PLB (9,14,15), and among these, diffuse large BCL (DLBCL) represents 54–92% (10,13,14,16). The frequency of T-cell subtypes is relatively high (24%) in the Chinese population compared with that in the Western population (9).

According to the Kiel classification (7), 45–78% of primary NHL of the bone are centroblastic and multilobulated (10,15,18). BCL-6 was positive in 30% of cases and strong p53 protein expression was observed in 11 out of 20 (55%) cases. A clonal B-cell process by immunoglobulin heavy gene rearrangement was also found in the majority of cases (13/18; 72%) (18). Another study has also demonstrated that p53 and Bcl-2 may be involved in the pathogenesis of PLB (19).

The literature has defined PLB in numerous different ways. Certain studies have only included patients with Ann Arbor stage I and II disease in the diagnosis of PLB (19,20), others have included patients with stage IV disease and yet more have included patients with involvement of the lymph nodes (3,21–23). At present, the following diagnostic criteria of PLB is widely accepted and includes the following conditions (6): i) Primary site of tumor origin in the bone marrow, with no other site indicating the existence of the lesion on physical or imagining examination; ii) no identification of lymphoma at any other site six months after the diagnosis of PLB; iii) the diagnosis must be confirmed by pathology and immunohistochemistry; and iv) malignant lymphomas, with the exception of PLB and secondary lymphoma of the bone, must be excluded.

A common complaint of patients with PLB is pain in the bones. However, as non-steroidal anti-inflammatory drugs may partly relieve these symptoms, PLB patients can be referred to rheumatologists and misdiagnosed with rheumatic diseases (24). Chronic myelitis, metastatic tumor of the bone and other primary bone tumors, such as osteosarcoma, must be excluded prior to determining the diagnosis.

This current case study presents a review of the radiological imaging of skeletal lymphoma with conventional radiographs, scintigraphic studies, computed tomography, MRI and FDG-PET-CT (25).

At the time of the initial radiograph, the results of plain X-rays are usually normal. A solitary lytic lesion near the end of a long bone, with a permeative or moth-eaten pattern of destruction, and a periosteal reaction can be observed in aggressive types, and this appearance is similar to that of metastatic lymphomatous involvement of the bone, osteosarcomas and Ewing’s sarcoma (26).

Radionuclide bone scans (^99m^Tc radionuclide imaging) show increased tracer uptake in 98% of patients, with markedly increased activity in 64% of patients (26), which is usually non-specific. However, bone scintigraphy of ^99m^Tc-methylene diphosphonate is a valuable tool in the staging of PLB. It detects multifocal involvement, which alters the prognosis and possible treatment (27).

CT is excellent in delineating cortical destruction, however, the features are usually non-specific. Within months of successful treatment, CT shows bone remodeling with a persistent architecture that is similar to that of Paget’s disease of the bone (28). The diagnosis of PLB can be indicated by CT and MRI, particularly when upon the observation of a large soft-tissue mass and abnormal marrow attenuation or signal intensity without extensive cortical destruction.

Compared with Ewing’s sarcoma or osteosarcoma, PLB shows significantly less frequent cortical abnormality, complete penetration, focal destruction and complete destruction on MRI (29,30). On T1-weighted MRI, the signal intensities in the lesion range between isointense and hypointense relative to the muscle, while on T2-weighted MRI, the signal intensities are varied and do not appear to just reflect the histological findings of intralesional vascularity or fibrosis (31). Following successful treatment, a rapidly decreasing tumor volume can be observed, with complete disappearance of the soft tissue component. Minor bone marrow signal abnormalities that have no clinical relevance may persist for up to two years (28).

PLB is usually shown as a hypermetabolic lesion on FDG-PET. PET-CT is a sensitive tool for accurately determining a response to therapy, particularly a complete response (CR). In cases of CR, PET scanning following treatment shows no hypermetabolic lesions, with a rapid decline in FDG uptake, in contrast to MRI or plain X-ray, which show a persistent bone lesion following a partial response (32). Newly developed lesions with rapid increase of FDG uptake, found by PET during the follow-up period in patients with a CR, are determined to be recurrence (33).

There is no standard therapy or guideline for PLB, as all previous literature studies have been retrospective. Therapy in general is multimodal and includes surgery, radiotherapy, chemotherapy and rituximab.

Although the likelihood of local control following the treatment of stage IE PLB is extremely high with radiotherapy alone, radiation alone in limited-stage disease has a poor five-year overall survival (OS) rate of ~45%, even when patients with apparently limited-stage disease have been carefully selected for treatment (27). Radiotherapy alone has not been found to improve survival, and 10-year survival has been shown to decrease in stage III patients. Therefore, radiation alone should only be used in patients with spinal cord compression (34), and more effective systemic regimens are required (35). It has been reported that the survival time is longer for patients treated with a combination of chemotherapy and radiotherapy than those treated with radiotherapy alone (16). Other studies have also found that chemotherapy combined with radiotherapy is superior to chemotherapy or radiotherapy alone, with five-year survival rates of 58–95 versus 70–78%, respectively (34,35).

PLB is sensitive to chemotherapy, however, Adriamycin- or anthracycline-based regimens have been confirmed to be successful in achieving excellent long-term, disease-free survival, particularly when followed by involved-field radiotherapy (13).

Progression-free survival (PFS) and OS times in patients with CD20-positive BCL have been markedly improved by adding rituximab to CHOP chemotherapy (37), however, the superiority of rituximab in PLB of DLBCL is controversial (27). A previous study showed marked improvements in the three-year PFS rate for PLB patients following the introduction of rituximab (88%) compared with those treated earlier without rituximab (52%) (17), and also provided evidence of improved survival with combined systemic therapy using rituximab and combination chemotherapy with CHOP (17). However, a retrospective analysis of patients with PLB demonstrated that the addition of rituximab to chemotherapy resulted in a non-significant trend toward a superior OS rate (38).

As surgery has not been found to improve OS or PFS, surgery is only indicated for prophylactic fixation of impending fractures or the treatment of pathological fractures or spinal cord compression.

PLB has an improved prognosis compared with other bone malignant tumors, such as osteosarcoma or secondary lymphoma of the bone (14). A younger age has also been identified as an independent predictor of survival (3). Heyning *et al* (3) found that patients who were >60 years old at the time of presentation exhibited poorer OS (76 vs. 37%) and a smaller progression-free period (58 vs. 28%) (14). Disease stage has also been found to have a significant effect on five-year OS. Patients with localized disease have statistically improved survival times compared with patients with systemic disease, and survival rates have been recorded as 90% for stage I and 41% for stage IV (10).

The international prognostic index (IPI) is a prognostic factor for PLB. A significant difference has been identified in the OS of patients low and low-intermediate versus high-intermediate IPI scores (P=0.0035), regardless of stage (27). In addition, younger patients with good IPI scores have a favorable prognosis (18).

Heyning *et al* (3) also found poorer survival times in patients with the immunoblastic subtype compared with the centroblastic mono/polymorphic or centroblastic multilobulated subtypes (P=0.015). This was also confirmed in a study by Lewis *et al* (4), in which statistically improved survival times were observed in patients with DLBCL with multilobulated nuclei (15).

A statistically significant difference has been identified in OS favoring the use of combined chemotherapy (with or without rituximab) and radiation compared with either modality alone (P=0.02) (27). Furthermore, the addition of rituximab has been found to result in a non-significant trend towards improved OS (P=0.11).

PLB is a distinct clinicopathological entity with a relatively homogeneous morphology and clinical behavior, and is usually of B-cell type. PET-CT is of great importance in evaluating CR, and patients with PLB treated with combined modality therapy have been found to exhibit a superior outcome compared with those treated by single modality therapy. In addition, younger patients with good IPI scores and localized disease have a favorable prognosis. The present PLB patient is a well documented case, who underwent full evaluation, received proper treatment, and had a good prognosis. This case demonstrates that PLB has an improved prognosis compared with primary lymphoma of other sites and that combined therapy may further improve outcome. However, future prospective studies must be performed in order to gain an improved understanding of the disease.
